# O hand, where art thou? Mapping hand location across the visual field during common activities

**DOI:** 10.1007/s00221-023-06597-7

**Published:** 2023-03-24

**Authors:** Joao Mineiro, Gavin Buckingham

**Affiliations:** Department of Public Health and Sports Sciences, University of Exeter, Exeter, UK

**Keywords:** Human hand, Visual field, Asymmetries, Manual object interaction, Machine learning

## Abstract

Humans employ visually-guided actions during a myriad of daily activities. These ubiquitous but precise manual actions rely on synergistic work between eye and hand movements. During this close cooperation between hands and eyes, the hands persist in sight in a way which is unevenly distributed across our visual field. One common assertion is that most hand actions occur in the lower visual field (LVF) because the arms are anatomically lower than the head, and objects typically rest on waist-high table surfaces. While experimental work has shown that humans are more efficient at reaching for and grasping targets located below their visual midline (Goodale and Danckert, Exp Brain Res 137:303–308, 2001), there is almost no empirical data detailing where the hands lie in the visual fields during natural hand actions. To build a comprehensive picture of hand location during natural visually guided manual actions, we analyzed data from a large-scale open-access dataset containing 100 h of non-scripted manual object interactions during domestic kitchen tasks filmed from a head-mounted camera. We found a clear vertical visual asymmetry with hands located in the lower visual scene (LVS) in more than 70% of image frames, particularly in ipsilateral space. These findings provide the first direct evidence for the established assumption that hands spend more time in the lower than in the upper visual field (UVF). Further work is required to determine whether this LVF asymmetry differs across the lifespan, in different professions, and in clinical populations.

**Supplementary Information:**

The online version contains supplementary material available at 10.1007/s00221-023-06597-7.

## Introduction

Humans use vision to guide the majority of their manual actions during daily activities. From preparing breakfast in the morning to hitting a ball while playing tennis, eyes and hands work closely together to achieve rapid and precise manual actions. During these activities of daily living, hands and the objects being manipulated are often under the scrutiny of the human eye (Land et al. 1999). In fact, hands and eyes work tightly together to the point that the former will often wait for the latter to guide and select subsequent targets (Pelz et al. 2001). When such close cooperation between hands and eyes is prevented, our visuomotor performance is negatively affected. For instance, when individuals avoid looking directly at a target, reaching accuracy is impaired (Henriques et al. 2003; Henriques and Crawford 2000). One of the consequences of such close connection between hands and eyes is that hands are often in sight. Despite the persistence of hands in our line of sight, the distribution of such persistence across our visual field is thought to be biased toward the lower half. One common assertion is that most hand actions occur in the LVF because the arms are anatomically lower than the head, and objects typically rest on waist-high table surfaces. The goal of this paper is to quantify the degree to which the hands operate in the LVF in typical daily tasks.

Although it is not yet known how long the hands actually spend in the LVF relative to the UVF, a significant body of experimental work has shown that humans are more efficient at reaching and grasping for targets located below their visual midline (Brown et al. 2005; Goodale and Danckert 2001; Graci 2011; Khan and Lawrence 2005; Krigolson and Heath 2006; Stone et al. 2019). The seminal work of Goodale and Danckert (2001) highlights a LVF functional advantage for visually guided skilled movements which is not solely explained by retinal factors. When they asked individuals to point as quickly and accurately as possible at a target which kept changing size, they found out that individuals were faster and more accurate when the target was located in the LVF. Further work since this initial observation found less variation in reaching and grasping movements when the target persisted in the LVF instead of better speed and accuracy. For instance, individuals applied less variation in their peak grip aperture when grasping in the LVF (Brown et al. 2005) and were more effective at adjusting limb trajectories in the late stages of pointing movements when such actions occurred in the LVF (Khan and Lawrence 2005). Similarly, Krigolson and Heath (2006) observed better endpoint precision in the LVF using a reaching task where target location alternated between constant and unexpectedly varied. This LVF performance bias has also been demonstrated in controlled functional tasks. When humans have the LVF occluded, they take longer to reach a glass of water placed on a desk and stand at a longer distance from that same desk/glass, compared to having full vision of the environment (Graci 2011). Interestingly, there is even evidence from sporting populations that this LVF bias is malleable. College-level basketball players (who spend a disproportionate amount of time with their hands in the UVF) also performed faster and more accurately in a reaching task when the target was located in the LVF, but this asymmetry was far smaller than in non-basketball players (Stone et al. 2019).

Support for a LVF specialization is not only found in behavioral work but also at the neuroanatomical level. For example, cone and ganglion cell density in the retinal region corresponding to the lower visual hemifield is nearly 60% higher than its contra vertical region (Curcio and Allen 1990). This vertical asymmetry continues as we move into intermediate levels of the visual system—for example, Schmidtmann et al. (2015) found that participants are more accurate at discriminating differences in object and face shapes in the LVF than the UVF. Specific higher cortical areas which reside anteriorly to the primary visual cortex and are implicated in controlling visually guided limb movements also show a functional preference for targets present in the LVF. Rossit et al. (2013) asked participants to grasp targets while fixating their gaze at one of four visual quadrants. The authors found a higher activation of the superior parieto-occipital cortex and the left precuneus (regions heavily involved in skilled manual actions) when participants reached for targets which were located in their LVF, compared to the UVF. Maltempo et al. (2021), using a visuomotor task involving pointing with the eye, the hand, or the foot, found not only a higher activation in the anterior superior parietal lobe but also in the dorsomedial parietal cortex when targets were presented in the LVF involved in visually guided upper and lower limb actions.

There is a strong body of evidence that actions performed in our LVF are supported by different neural structures than those in the UVF. Furthermore, there are clear anatomical and contextual reasons to assume that the hands spend more time in the lower than in upper visual field. However, this assumption has received no empirical support, and the degree of this putative asymmetry has not been quantified. Capturing the spatial preferences of hands from the individual’s point of view in uncontrolled environments could provide further insight into the development, acquisition and impairment of human visuomotor skills. Furthermore, mapping hand location during natural hand movements could validate a body of knowledge on visually guided hand movement efficiency which is predominantly built on controlled environment findings. Ecologically robust findings of hand spatial preferences during visually guided movements are not only relevant for fundamental research but also have tangible applications in clinical and industry contexts. For instance, there are clear applications to support the current shift to tele health services for stroke survivors (Laver et al. 2020) and for individuals with musculoskeletal conditions (Murray et al. 2021). The spatial preferences of the hands can be translated to rehabilitation monitoring or workstation planning by remotely assessing and tracking the progress of individuals in their own environment during meaningful hand-based tasks.

Most research examining visual field preference uses eye tracking or constrained visual fixation to quantify the visual field of actions and stimuli. A visual scene (VS) is the head-centric visual space experienced by the individual when situated in a specific context (Intraub 2012). Gaining access to the statistical regularities of the VS can provide ecologically robust understanding of why humans are more efficient at performing hand movements in certain spatial areas in contrast to more ‘neglected’ ones. The difficulty of labeling large volumes of images, which is often performed manually by researchers (Niehorster et al. 2020), has been delegated to machine learning-based image classification models (Shan et al. 2020) yielding computational models which are accurate at identifying and labeling objects in image and video. These advances in image labeling, together with the easy access and application of head-mounted cameras have produced several datasets on hand location and object interaction status which are openly accessible online to other fields of fundamental and clinical science (Damen et al. 2018).

In this paper, we show how we examined a large-scale open-access dataset of naturally occurring (i.e. non-scripted) manual object interactions from a domestic kitchen setting filmed from a head-mounted camera to determine where in space the hands are located during real-world tasks. From this dataset, we were able to extract the positions of the hands in the visual scene and build a comprehensive picture of where, in the viewers’ head-centered close space, manual actions typically occur.

## Methods

### Dataset

We examined VS asymmetries during manual actions in a large-scale open-access dataset named EPIC-KITCHENS-100 (Damen et al. 2022, 2018). The dataset is composed of 100 h of video recordings of naturally occurring (i.e. non-scripted) manual object interactions (e.g. making a cup of tea or slicing cake). To make this dataset, over 20 million frames of first-person perspective were recorded using a head-mounted camera by 37 participants while in their own domestic kitchens located in 4 different international cities. In brief, participants were asked to fit the GoPro head-mounted camera themselves and to check battery life and viewpoint using the GoPro Capture app installed on their phones. Viewpoint was determined by aligning the camera in a way that the participant’s stretched hands were located in the centre of the screen. The cameras were set to linear field of view, recording at 59.94 frames per second and 1920 × 1080 resolution. Some participants changed video and screen resolution; however, this only affected 1–2% of the videos (Damen et al. 2018).

The analysis of visual scenes rather than visual fields was underpinned by a number of assumptions. We assumed that the collected image fields (i.e., the head-mounted camera field of view) coincided with the Cartesian centre of the VS. We are aware that the eyes follow the object under manipulation before the hand is in contact with the object (Land et al. 1999) and eye movements do not always coincide with head movements (Pelz et al. 2001), which may have added variance to our results. However, experimental work also shows that head position modulates visual perception with humans performing better during visual identification tasks when their heads are aligned with target position, irrespectively of head–eye alignment (Nakashima and Shioiri 2015). We also assumed that hands were correctly centered in the image Cartesian middle coordinates during head-mounted camera setup. The relevance of this assumption is based on experimental work showing that the eyes tend to fixate the index finger object contact point while guiding the hand in engaging with objects (Cavina-Pratesi and Hesse 2013; Voudouris et al. 2016). If visual perception and attention are improved by aligning the head with hand interaction targets and if fixations follow the index finger contact point, we assumed that by centering these two factors to the centre of visual scene participants would be often looking to an approximation of the visual field centroid. This assumption is also supported by the work of Foulsham and colleagues (2011) in which the use of head-mounted eye tracking showed that adults spend most of the time fixating the centre of their visual field when walking around a natural scene on their way to get a coffee. We further explore the impact of these assumptions on our findings in the ‘Discussion’ section.

**Fig. 1:**
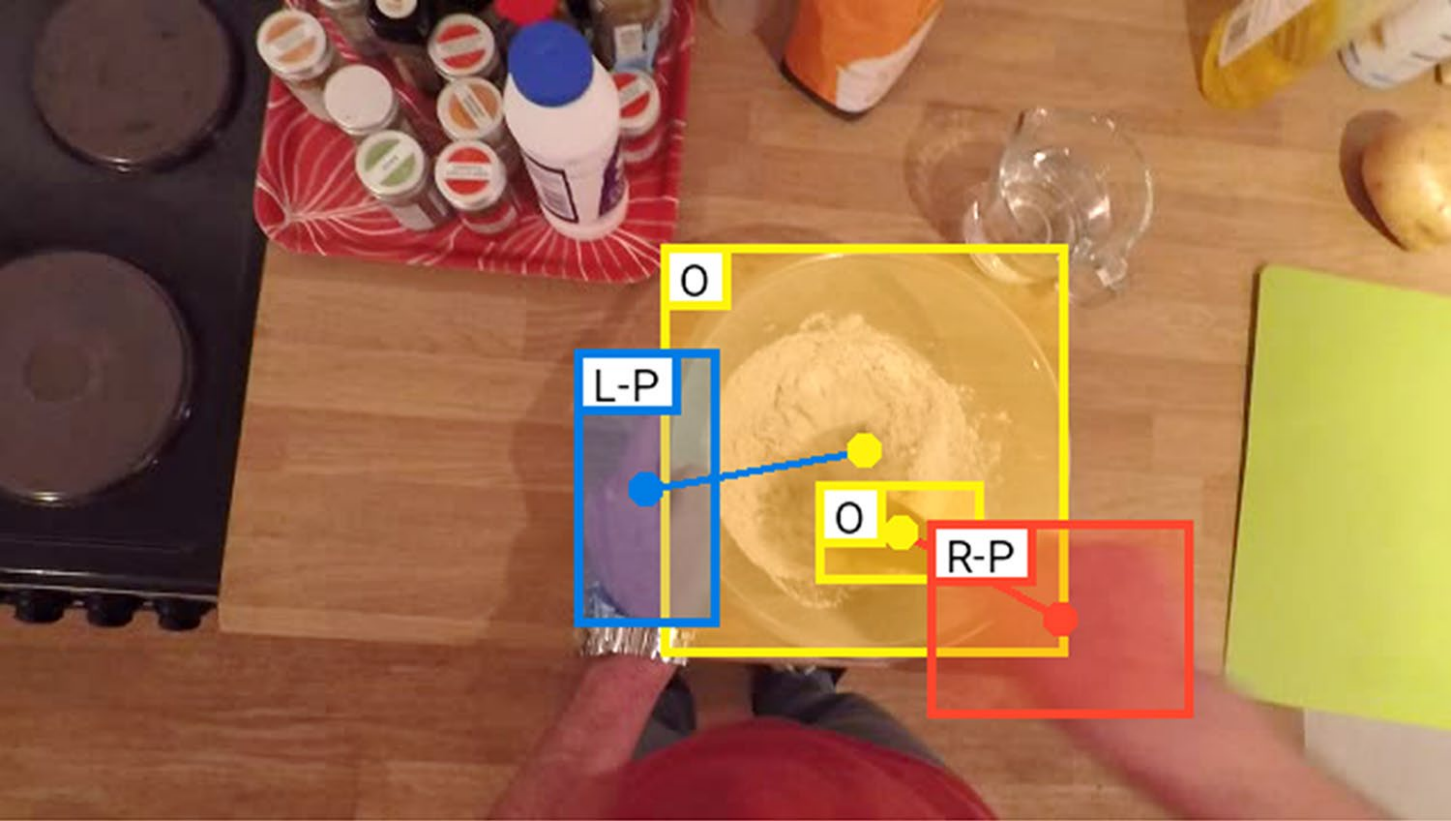
An example of a video frame and respective hand and object boundary boxes after classification by the model. The Cartesian coordinates were processed to determine whether the hand is above or below the visual horizontal midline correspond to the centre of the box (the blue dot in the blue box and the red dot in the red box). From (Price and Ray 2020)

To conduct our analysis, we processed the Hand–Object automatic annotations from the EPIC-KITCHENS-100 dataset (Damen et al. 2022). These annotations were produced using a hand–object interaction classification model (Shan et al. 2020). The authors of EPIC-KITCHENS-100 (Damen et al. 2022) reported that they trained the model using 100 K images from YouTube together with 42 K images from other egocentric datasets (Damen et al. 2018; Li et al. 2015; Sigurdsson et al. 2018). The original authors made sure that 18 K of the 42 K images were from their own videos (Damen et al. 2018). The model by Shan et al (2020) labels hand–object interactions in each frame. With regards to hand–object classification, the model identifies whether one or both hands are present in each frame, determines hand laterality, hand and object spatial location, and draws a bounding box (‘bbox’) around hand and object. An example of a ‘bbox’ can be found below (Fig. 1) when the original authors rendered the hand–object interaction classification.

The same model also classified hand–object interaction state—whether the hand is interacting with a ‘portable’ object, a ‘stationary’ object, in ‘no contact’ with any object or in ‘self-contact’ with the other hand. ‘Portable’ objects included small objects which were required to perform kitchen tasks such as small kitchen appliances, crockery and different food and drink items. ‘Stationary’ objects included larger and static kitchen appliances or furniture found in the kitchen such as fridges, kitchen tops or ovens. ‘No contact’ state included actions involving hand free roaming as well as during reaching for objects or reaching for the other hand. Hand ‘self-contact’ includes actions involving one hand touching the other such as washing, drying or brushing the hands. We were unable to include a full list of actions classified by hand–object interaction state as these data are made only partially available by the authors.

All annotations from the classification model were saved in a python ‘object’ which can be accessed using a supporting python library. We developed a script to extract the following data from the annotation files: participant number, video number, frame number, hand spatial location and hand–object interaction state. With regards to hand spatial location, we only collected hand ‘bbox’ centre y coordinate, ‘bbox’ left coordinate, ‘bbox’ width, frame width and height. Using this information, we were able to determine the 2D spatial centre coordinates for each hand across time. Frames were 456 × 256 pixels; hence, the horizontal centre was at 228 pixels and the vertical centre at 128 pixels. We were able to determine the ‘bbox’ centre y coordinate directly from the annotations but had to calculate the ‘bbox’ centre x coordinate by adding half the ‘bbox’ width to its left coordinate.

### Data availability

We accessed the Hand–Object automatic annotations from the EPIC-KITCHENS-100 dataset files using the supporting python library provided by the dataset authors and hosted on an online repository (Price and Ray 2020; available here: https://github.com/epic-kitchens/epic-kitchens-100-hand-object-bboxes). More information on the image classification model and corresponding library can be found in Shan et al. (2020). A total of 703 Pickle files were analyzed through the Google Colab online platform. In the interest of the reproducibility of our work, we provide the respective Jupyter Notebooks we developed in Google Colab which can be used to follow the steps we took to reach our findings (https://osf.io/uwe9k/).

### Dataset analysis

The resultant dataset required two transformations. Every frame containing both left and right hands had to be duplicated as we could only process the location of one of the hands at the time. The spatial coordinates provided for the ‘bbox’ are relative to the origin coordinates (0, 0) of the frames. However, the origin (0, 0) of the frame corresponds to the top left corner rather than the Cartesian origin of a plot—the bottom left corner. For that reason, we converted every y-centre coordinate to Cartesian so the finding could be plotted. We also developed a second script to calculate the total number, and respective proportion, of frames across vertical and horizontal visual hemi scenes, and visual scene quadrants. This second script processed data by both hands, each of the hands and also by handedness.

Participant handedness information was not collected in the original dataset. However, to estimate handedness, we downloaded and watched the video footage for each participant, available in Damen et al. (2022). We then used items from the Edinburgh Handedness Inventory (Oldfield 1971) to determine participant handedness, through four activities (the use of scissors, spoon and knife with and without a fork) performed in the videos. We assigned a point to the hand employed to perform one of the activities aforementioned. The hand side with the highest score determined participant handedness.

### Statistical analysis

Data were analyzed using the Pingouin statistics package for Python 3 (Vallat 2018) inside a Google Colab notebook. We set statistical significance at *α* = 0.05. Effect size was reported as Cohen’s dz. Statistical differences between proportion of hands in the various quadrants, hemi scenes, and during hand interactions were tested using paired samples *t *tests. In the interest of the reproducibility of our analysis, we provide the respective Jupyter Notebook we developed in Google Colab which can be used to follow the steps we took to reach our findings (https://osf.io/uwe9k/).

## Results

### Descriptive findings

All video annotations from the 37 participants in the original EPIC-KITCHENS-100 dataset were included in the study. In total, 703 automatic annotation files and respective 20,076,005 frames were processed. Subsequently, we identified 15,761,306 frames which contained at least 1 hand. To analyze single hand location, we ended up with a total of 31,300,201 frames (for information about how this value was arrived at, see Methods). From the total number of frames analyzed, we found a higher number of frames containing right hands (16,123,924) than those containing left hands (15,176,277). The majority of participants were classified as right handed (*n* = 32), with the remaining five participants classified as left handed.

Despite the fact that the main aim of the paper was to answer how hand location is distributed across vertical hemi VS, we also examined hand location across VS quadrants and during different hand interactions. We found that hands spent most of the time interacting with portable objects (83.2%), or not in contact with anything (12.7%). The hands were in contact with a stationary object 2.9% of the time, and were in contact with the contralateral hand 1.2% of the time.

### Hand location across image vertical hemi space

**Fig. 2:**
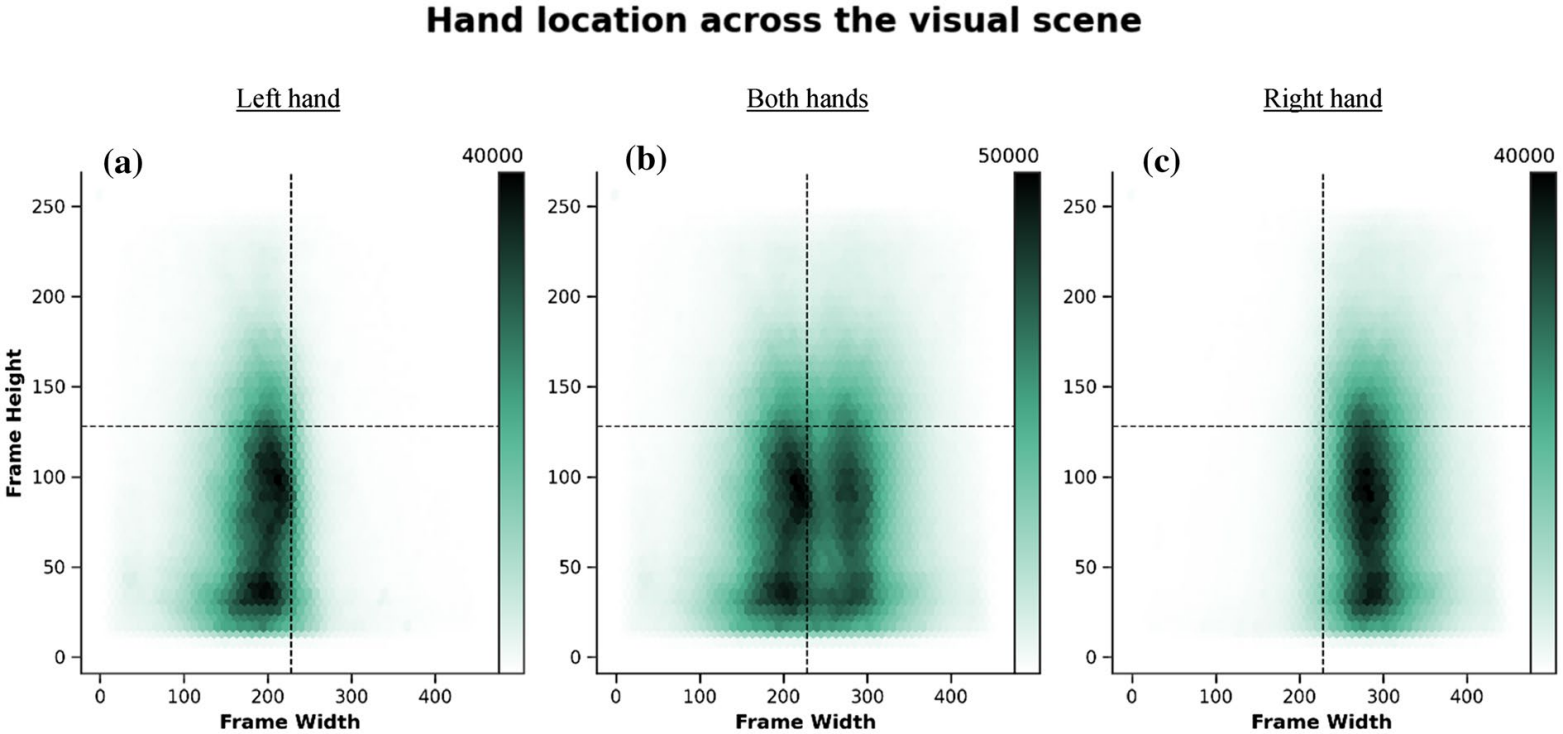
Hex plots showing the overall distribution of hand location (Table 1); **a** for the left hand only, **b** irrespectively of hand (i.e., both left and right hands, for unimanual and bimanual movements) and **c** for the right hand only, across the image space. Total number of frames analyzed = 31,300,201. The darkness of the color reflects the density of the coded hand (attached color bar indicates frame density across the visual scene), which are similarly reflected in the horizontal and vertical axis histogram bars on the top and side of the image

We found a higher proportion of frames where hands were located in the LVS, when compared to the UVS (Table 1 and Fig. 2). This marked vertical asymmetry in hand location was also present when we explored the dataset by hand side. We found that both left and right hands were present more often in the LVS than in the UVS (Table 1 and Fig. 2). These values were similar in both left handers and right handers (plots and tables for these data by hand dominance can be found in the supplementary materials).

**Table 1:** Frame proportions across vertical hemi scenes, UVS and LVS (visually presented in Fig. 2)

Hand location across vertical hemi scene (Frame %)			
		UVS	LVS
Total frames	Either hand	24.0	76.0
	Left hand	21.4	78.6
	Right hand	26.5	73.5

**Fig. 3:**
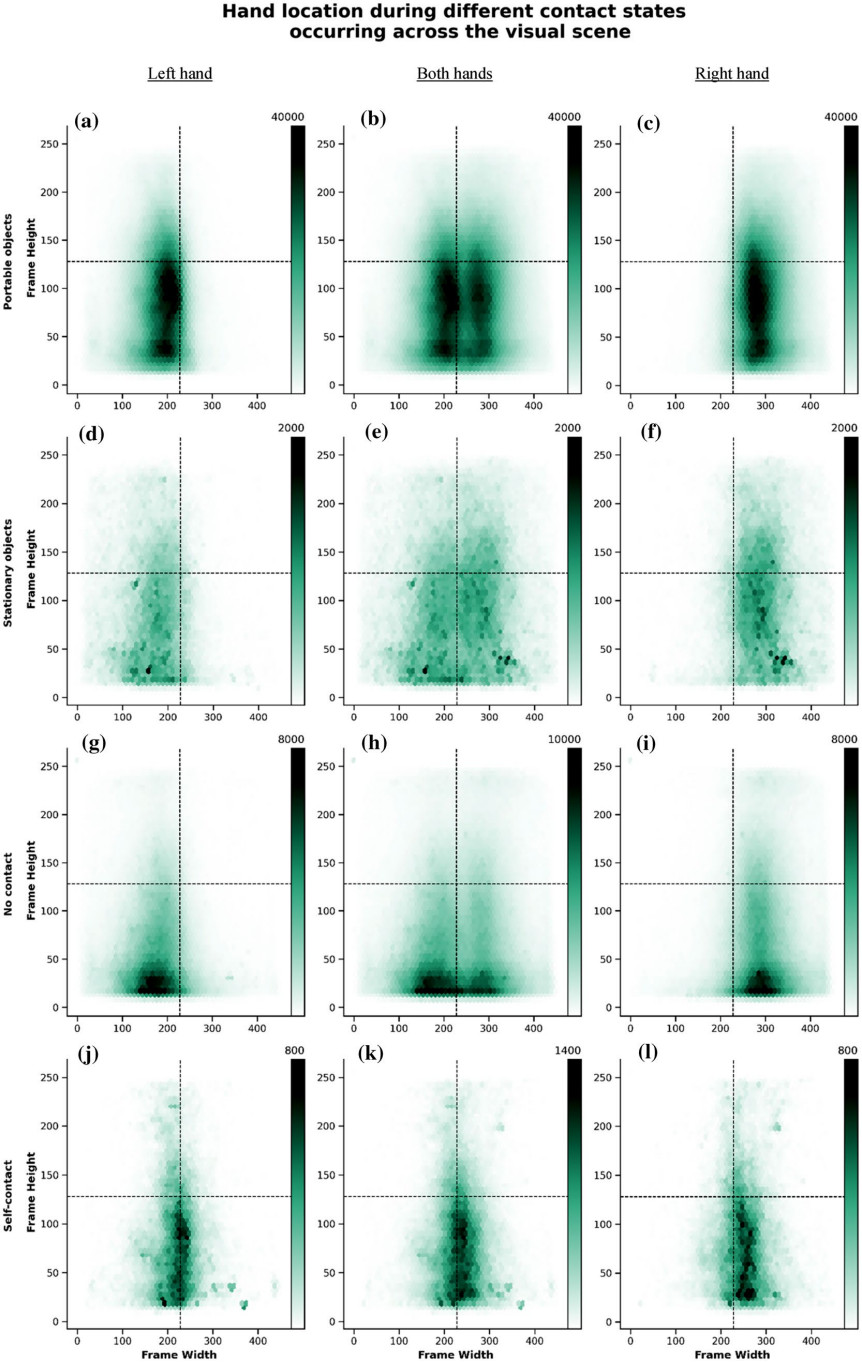
Hex plots representing the overall distribution of hand location during the different contact states across the visual scene (Tables 2 and 4). In the first row, the three-panel figure shows the overall distribution of hand location during portable object interaction, **a** for the left hand only, **b** irrespectively of hand used (d, i.e., both left and right hands, for unimanual and bimanual movements); and **c** for the right hand only, across the image space; total number of frames analyzed = 26,054,826. In the second row, the three-panel figure shows the overall distribution of hand location during stationary object interaction, **d** for the left hand only, **e** irrespectively of hand used **d** ( i.e., both left and right hands, for unimanual and bimanual movements) and **f** for the right hand only, across the image space; total number of frames analyzed = 901,915. In the third row, the three-panel figure shows the overall distribution of hand location during no object contact, **g** for the left hand only, **h** irrespectively of hand used **g** (i.e., both left and right hands, for unimanual and bimanual movements) and **i** for the right hand only, across the image space; Total number of frames analyzed = 3,968,863. On the forth row, the 3-panel figure shows the overall distribution of hand location during hand self-contact, **j** for the left hand only, **k** irrespectively of hand used **j** (i.e., both left and right hands, for unimanual and bimanual movements) and **l** for the right hand only, across the image space; total number of frames analyzed = 374,597. The darkness of the color reflects the density of the coded hand (attached color bar indicates frame density across the visual scene), which are similarly reflected in the horizontal and vertical axis histogram bars on the top and side of the image

We also found that hands were more often located in the LVS during all object interaction states, when compared to the UVS (Table 2 and Fig. 3). Similarly, both left and right hands were present more often in the LVS than in the UVS during object interactions (Table 2 and Fig. 3). We found similar proportions in both left and right handers (plots and tables for these data by hand dominance can be found in the supplementary materials).

**Table 2:** Frame proportions during the different contact states occurring across vertical hemi scenes, UVS and LVS (visually presented in Fig. 3)

Hand location during object interaction across vertical hemi scenes (Frame %)			
Portable objects			
		UVS	LVS
Total frames	Either hand	25.0	75.0
	Left hand	22.6	77.4
	Right hand	27.2	72.8

No-contact			
		UVS	LVS
Total frames	Either hand	17.0	83.0
	Left hand	13.8	86.2
	Right hand	20.7	79.3

Stationary objects			
		UVS	LVS
Total frames	Either hand	29.1	70.9
	Left hand	24.9	75.1
	Right hand	32.9	67.1

Self-contact			
		UVS	LVS
Total frames	Either hand	19.5	80.5
	Left hand	19.5	80.5
	Right hand	19.5	80.5

### Hand location across image quadrants

When we looked at hand location irrespective of hand side and hand interaction, we found that hands were more often located in the lower quadrants. The upper left quadrant was the least visited area of the visual space (Fig. 2 and Table 3). We found similar values in left handers and right handers. Plots and tables for these data by hand preference can be found in the supplementary materials.

**Table 3:** Frame proportions across visual scene quadrants (visually presented in Fig. 2)

Hand location across visual scene quadrants (Frame %)					
		UL	LL	UR	LR
Total frames	Both hands	10.0	35.6	14.1	40.3
	Left hand	18.3	65.9	3.1	12.7
	Right hand	2.1	7.1	24.4	66.3

Interestingly, when we looked at hand side irrespective of hand interactions (Fig. 2 and Table 3), we found that each hand is more often present in its ipsilateral visual scene and almost neglects locations in the contralateral UVS with similar values in both left handers and right handers. Plots and tables for these data by hand side can be found in the supplementary materials.

We found that hands were also more often located in the lower quadrants and the upper left quadrant was the least visited visual space during the different contact states (Fig. 3 and Table 4). Similar results were found in left handers and in right handers. Plots and tables for these data by hand preference can be found in the supplementary materials.

**Table 4:** Frame proportions during object interactions across visual scene quadrants for all participants (visually presented in Fig. 3)

Hand–object interactions across visual scene quadrants (Frame %)					
Portable objects					
		UL	LL	UR	LR
Total frames	Both hands	10.3	40.0	14.7	40.4
	Left hand	19.4	65.2	3.2	12.2
	Right hand	2.0	6.4	25.2	66.4

No-contact					
		UL	LL	UR	LR
Total frames	Both hands	7.2	43.0	9.8	40.0
	Left hand	11.6	72.2	2.2	14.1
	Right hand	2.4	10.5	18.3	68.8

Stationary objects					
		UL	LL	UR	LR
Total frames	Both hands	12.1	34.6	17.0	36.3
	Left hand	21.7	64.5	3.2	10.6
	Right hand	3.4	7.6	29.5	59.5

Self-contact					
		UL	LL	UR	LR
Total frames	Both hands	9.4	34.5	10.1	46.0
	Left hand	12.9	47.2	6.6	34.3
	Right hand	5.7	22.1	13.9	58.4

### Statistical findings

To quantify for significant differences between the various quadrants and hemi scenes, we calculated the respective within-subject frame averages for all the participants (*n* = 37) and then conducted paired samples *t *tests with an alpha threshold of 0.05. For the comparisons tested in Table 6, a Bonferroni-corrected alpha threshold of 0.00625 (0.05/8) was used as the threshold for statistical significance. We also calculated 95% confidence intervals for the mean difference and effect sizes in the form of Cohen’s dz.

We found significant differences between the proportions of frames with hands located in the lower and upper hemi scenes as well as between hands (Tables 5 and 6). Hands were more often in the lower hemi scene than on the upper hemi scene (74.3% vs. 25.7%, *p* < 0.001; Table 5). Interestingly, the left hand was more often located in the lower hemi scene than the right hand (77.1% vs. 71.6%, *p* < 0.001; Table 6). As a consequence, the right hand was more often located in the upper hemi scene than the left hand (28.4% vs. 22.9%, *p* < 0.001; Table 6).

**Table 5:** Statistical differences between hemi scenes: mean frame percentages for each hemi scene, paired samples *t *test score, *p* value for the mean difference, 95% confidence interval for the difference and effect size (Cohen’s dz)

Statistical differences between hemi scenes						
Hand location	Mean hand % Upper scene	Mean hand % Lower scene	t(36)	*p*	CI95%	Cohen’s dz
Vertical hemi scenes	25.7	74.3	− 10.525	< 0.001	− 57.95, − 39.22	3.461

	Mean hand % Ipsilateral	Mean hand % Contralateral	t(36)	*p*	CI95%	Cohen’s dz
Horizontal hemi scenes	88.2	11.8	65.819	< 0.001	74.13, 78.84	21.641

**Table 6:** Statistical differences between the proportion of frames containing the left or the right hand in the various hemi scenes and quadrants: mean frame percentages for each hand, paired samples *t *test score, p value for the mean difference, 95% confidence interval for the difference and effect size (Cohen’s dz)

Statistical differences between the left and the right hands						
Hand location	Mean % Left hand	Mean % Right hand	t(36)	*p*	CI95%	Cohen’s dz
Lower hemi scene	77.1	71.6	5.524	< 0.001	3.52, 7.6	0.386
Upper hemi scene	22.9	28.4	− 5.524	< 0.001	− 7.6, − 3.52	0.386
Contralateral hemi scene	16.2	7.6	4.440	< 0.001	4.65, 12.46	1.242
Ipsilateral hemi scene	83.8	92.4	− 4.440	< 0.001	− 12.46, − 4.65	1.242
Contralateral upper quadrant	3.3	1.8	3.204	0.003	0.53, 2.35	0.817
Ipsilateral upper quadrant	19.6	26.6	− 6.612	< 0.001	− 9.14, 4.85	0.515
Contralateral lower quadrant	12.9	5.8	4.400	< 0.001	3.83, 10.39	1.166
Ipsilateral lower quadrant	64.2	65.8	− 0.916	0.365	− 5.0, 1.89	0.115

We also found significant differences between the proportions of frames with hands located in the ipsilateral and contralateral hemi scenes as well as between hands (Tables 5 and 6). Hands were more often located in the ipsilateral hemi scene than in the contralateral hemi scene (88.2% vs. 11.8%, *p* < 0.001; Table 5). Interestingly, the left hand was more often located in the contralateral hemi scene than the right hand (16.2% vs. 7.6%, *p* < 0.001; Table 6). Correspondingly, the right hand was more often in the ipsilateral hemi scene than the left hand (92.4% vs. 83.8%, *p* < 0.001; Table 6).

We found significant differences between hands across quadrants (Tables 5 and 6). The left hand was more often located in its contralateral upper quadrant than the right hand (3.3% vs. 1.8%, *p* = 0.003; Table 6). The right hand was more often used in its ipsilateral upper quadrant than the left hand (26.6% vs. 19.6%, *p* < 0.001; Table 6). The left hand was more often used in its contralateral lower quadrant than the right hand (12.9% vs. 5.8%, *p* < 0.001; Table 6). No significant differences were found between how often either hand visited the ipsilateral lower quadrant (65.8% vs. 64.2%, *p* = 0.365; Table 6).

## Discussion

### Strengths and weaknesses of our analysis

We analyzed a large-scale open-access dataset of natural hand movements during domestic kitchen tasks from 37 individuals. We found a clear bias for the hands to spend the majority of their time in the lower half of the visual scene. These results address the lack of real-life data on the spatial regularities of hand actions during visually guided tasks in the literature.

The analyzed dataset was collected using a head-mounted camera which was set up by participants themselves, according to instructions provided by the authors of the original dataset. The camera angle of view will not correspond exactly to eye level and will follow head movements rather than combined head and eye movements. Furthermore, each video frame is 456 by 256 pixels which creates a rectangular field of view but guarantees a landscape perspective of what the participant is observing during their activities. The nature of the captured field of view will not necessarily correspond to the human field of view but we feel it is a close approximation. Potential differences in people’s height might have changed the scale of the scene analyzed. These scene scale differences may be ameliorated by the fact that participants recorded themselves in their own kitchen, which might be adapted to individuals’ height and arm length. Furthermore, head-centered viewpoints of hands might not have always been aligned with the centroid of the collected image fields. Nonetheless, experimental work has shown that humans perform better during visual identification tasks when their heads are aligned with target position, irrespectively of head–eye alignment (Nakashima and Shioiri 2015). The interpretation of our results should take these factors into consideration—despite the richness of naturally occurring hand movement, the use of the head-mounted camera will have had added unknown variance to the data captured.

In the original dataset collection, participants were allowed to pause the video and narrate what they had been doing. This process may have caused participants to perform their movements less naturally than they otherwise might have. Moreover, a large number of the activities performed involved handling sharp objects such as knives or scissors as well as handling hot food over a heat source, therefore demanding close monitoring of their hands in the LVS. Kitchens are ultimately environments developed by, and for, humans, and hence afford more actions in the LVS. Following the constraint that arms are anatomically lower than the head, kitchen furniture is mostly available below, or at the same level as the centre of the head and portable objects typically rest on waist-high table surfaces. Nevertheless, kitchen-based tasks have long been a subject of study in neuropsychological research as these tasks relate well to a variety of cognitive functions including visuospatial skills (Yantz et al. 2010). Furthermore, the ubiquitous nature of these daily tasks and the use of both single and bi-manual unscripted actions across the entire visual scene make them ecologically robust and representative of healthy populations. Kitchen-based tasks are also widely used in neurological rehabilitation, both in the assessment and treatment of individuals with acquired brain injury (Mohapatra and Kulnik 2021) and are considered meaningful and highly valued by these same individuals (Bigelius et al. 2009). Moreover, the fairly low ambulatory demands of kitchen-related tasks also make them a good activity to contrast to other activities involving visually guided manual actions combined with ambulation in wider spaces such as in sports contexts or factory environments. We recommend that these different contexts are explored in future research.

Participant handedness was estimated based on observation from the use of a qualitative tool (Oldfield 1971) rather than through the administration of a participant questionnaire. Our estimates are less robust and the respective findings should be taken with care. Nonetheless, participant handedness proportion is in line with the general population—left-handed individuals corresponding to only 10% of the world population (Sha et al. 2021).

Our overall analysis examined over 31 million images of hands across 100 h of video footage. Size sample and respective data analysis meant that it was virtually impossible to manually check the findings of both the hand classification model and our analysis of the annotations. The hand classification model (Shan et al. 2020) has been trained using several large-scale datasets and showed strong external validity. The authors have trained this model using different large-scale image datasets and reported a 90.4% precision in identifying a hand in the image, 88.4% precision of identifying hand side, and 73.2% identifying the hand contact state (see Methods for reference to works where this was done).

The results of our analysis align with the literature on the topic and the methods used to reach such findings have been made available in the supplemental section. We wanted to guarantee that the methodological process was transparent and reproducible. We were not involved in any process related to the collection and analysis of the original dataset and have no affiliation to the research team responsible for the original dataset. We provided a detailed step-by-step description developed in Google Colab in the form of a Jupyter notebook with description of the steps taken and Python code (version 3.7.13) to run the analysis we applied on the dataset (available in supplementary materials and here: https://osf.io/uwe9k/).

We think that the methods used in the paper can be transferred to fundamental and clinical research. In the first instance, access to large-scale datasets of image classification of natural behavior is on the rise. Computer vision research labs continue to develop machine learning models and make them open access. Further analysis of such ecologically robust datasets can provide further knowledge on how visuomotor skills are developed, can be improved, and become impaired. We also hope the current work has encouraged the use of computer vision-based methodologies as a potential tool to study human behavior in a more ecologically robust way. Such methodologies can also be employed to test novel ways to help remote rehabilitation of individuals in their own environments. Nowadays, head-mounted cameras are easily accessible at reduced cost. Furthermore, image classification machine learning models, such as the one used to generate the dataset analyzed, are open access and can be run in cloud-based coding environments. Nevertheless, we are aware that these methods still present some barriers to their use. Specifically, the use of machine learning models requires computer coding skills and knowledge of machine learning model training. Step-by-step guides on how to train and use these models, as is the case of Shan’s model, are often available.

### Hand location during visually guided manual actions

We analyzed a large-scale dataset of manual object interactions during domestic kitchen-based tasks to build a comprehensive map of hand location during naturally occurring visually guided manual actions. This open-access dataset (Aldamen et al. 2020) represents 100 h of video footage captured from a head-mounted camera and contains over 20 million frames. We found that hands were present in the visual scene for most of the time (78.51% of the frames analyzed) but unevenly distributed across the visual space. We found that hands were located more often in the lower half of the visual scene and that this asymmetry was present irrespective of hand, hand dominance, and hand–object interaction state. Interestingly, the left hand was on average more often located in the lower hemi scene while the right hand was on average more often located in the upper hemi scene. These results provide empirical data in support of the common assertion that hands spend most of the time in the line of sight, predominantly in the LVF. Furthermore, the findings also add to work in controlled settings showing that humans are more efficient at reaching and grasping for targets located below our visual midline (Brown et al. 2005; Goodale and Danckert 2001; Graci 2011; Khan and Lawrence 2005; Krigolson and Heath 2006; Stone et al. 2019).

Interestingly, when we analyzed data by visual quadrants, we also found that each hand spent longer in their ipsilateral LVS irrespective of hand side, hand dominance, and hand–object interaction state. Our results mirror the findings from lab-based work demonstrating that humans are more efficient reaching for targets on the same side of the body as the limb used as opposed to reaching for targets on the contralateral side of the body (Barthélémy and Boulinguez 2002; Fisk and Goodale 1985; Hodges et al. 1997; Kim et al. 2011; Le and Niemeier 2014). This ipsilateral performance advantage has been linked to the effector side rather than to the location of the target (Fisk and Goodale 1985) and can be explained by the biomechanical characteristics of the movement itself (Carey et al. 1996). Slower and less accurate visually guided manual actions due to neuroanatomical and biomechanical constraints will instead result in neglected areas of our visual field. Lab-based experimental work by de Bruin et al. (2014) found a physiological ‘neglect’ of the upper left quadrant during 3D block construction tasks where participants had to reach and grasp for blocks available in equal numbers across a tabletop separated into quadrants. Despite having equal access to the same kind of objects in all quadrants of the tabletop, participants predominantly pick blocks from the lower right quadrant and leave the upper left quadrant for later in the task, irrespectively of individual handedness and hand side. We also found a specific visual quadrant neglect but in our dataset, this neglect was always in the contralateral UVS irrespectively of hand, handedness, and object interaction. In the overall sample, the right hand neglected the upper left quadrant on average nearly twice as often when compared to the left hand. Some explanations for the contrast between our findings and those of de Bruin et al. (2014) may be related to specific task contextual aspects, namely the fact that our findings reflect a more varied repertoire of hand actions performed in a larger 3D space, where different objects will be located in specific areas of the visual scene and will afford different movements in detriment of others based on spatial and biomechanical constraints. Our findings may have practical implications in the planning and design of skill acquisition, improving performance and developing recreational experiences. Designing physical and virtual environments which afford optimal manual movements may improve task performance, rehabilitation engagement, or simply improve our working and recreational experience.

### External validity of our findings

It must be acknowledged that it is unclear how much the findings from this paper would generalize to the whole repertoire of human manual activities; therefore, we advise caution in interpreting our findings outside the context of kitchen-based visually guided manual actions. Different proportions of hand–object interactions, context specificity of the dataset, and the absence of data on specific actions will have influenced the external validity of our findings. We found a much larger proportion of frames where hands are interacting with kitchen portable objects in comparison to other hand–object interactions. This predominance of portable object interaction data, combined with the fact that kitchen-based activities are often bimanual and tool based, makes our findings more transferable to tool-use activities. Kitchens are often small contained environments designed in a way that there is a predominance of objects below or at the same level as our eyes. The context specificity of kitchen-based activities will have likely influenced hand location. The dataset we analyzed does not provide specific information regarding the different employed actions during kitchen-based activities. The absence of information on the nature of the actions employed, their frequency and duration, makes it impossible to draw stronger conclusions about hand use. For instance, the second largest sample of frames represented hand locations during no contact with either other object or the contralateral hand. We are unable to differentiate between hand location during actions where the hand is reaching for objects and actions when the hand is not involved in any object interaction. Further research would benefit from developing and analyzing similar datasets in different professional and recreational activities, environmental contexts, and clinical populations. Advances in machine learning models will no doubt also enhance the capacity to recognize a broader range of actions and yield richer datasets.

## Conclusion

We have shown that, in the context of typical kitchen-based activities, hands were frequently in sight but their presence across the visual scene was not evenly distributed. Hand presence during visually guided manual object interactions was more often located in the lower and ipsilateral visual hemi scenes. Furthermore, hands revisited the upper and contralateral visual quadrant far less than the remaining visual space. Our findings are consistent with experimental work in controlled environments showing ipsilateral LVF advantages in a number of kinematic parameters. Further research should compare natural hand movements from different age, professional, recreational, and clinical populations.

## Supplementary Information

Below is the link to the electronic supplementary material.

Supplementary file1 (DOCX 6810 KB)

## Data Availability

Generated and analysed datasets supporting our findings of this study are available in OSF with the identifier https://osf.io/uwe9k/.
