# Supplementary material for: O hand, where art thou? Mapping hand location across the visual field during common activities
Source: Exp Brain Res. 2023 Mar 24;241(5):1227–39. doi: 10.1007/s00221-023-06597-7 (PMC10130124; doi:10.1007/s00221-023-06597-7)
Supplement: Supplementary file 1 — Supplementary file1 (DOCX 6810 KB) [file 221_2023_6597_MOESM1_ESM.docx]

**Supplementary materials – remaining plots and tables**


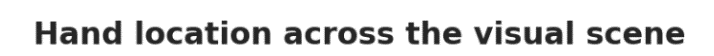


Left hand

Both hands

Right hand

**Left handers**


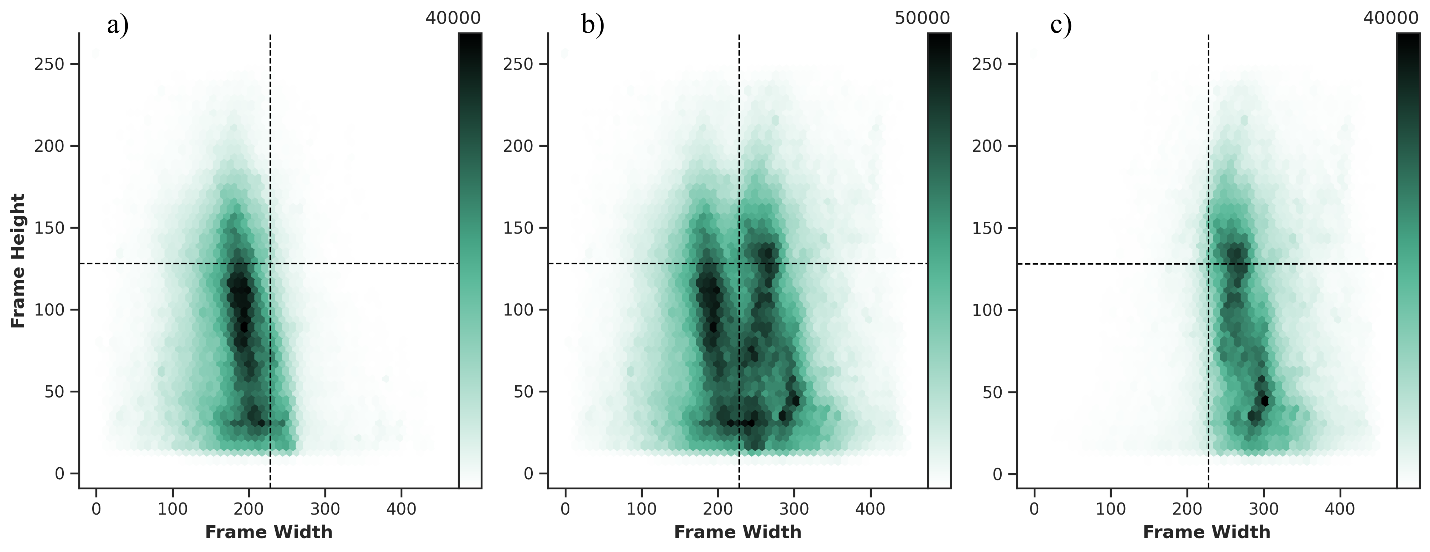


**Figure 1** – Hex plots showing the overall distribution of hand location for participants who were designated as left hand dominant (Table 1); (a) for the left hand only, (b) irrespectively of hand (i.e., both left and right hands, for unimanual and bimanual movements) and (c) for the right hand only, across the image space. Total number of frames analysed = 4,333,315. The darkness of the colour reflects the density of the coded hand (attached colour bar indicates frame density across the visual scene), which are similarly reflected in the horizontal and vertical axis histogram bars on the top and side of the image.


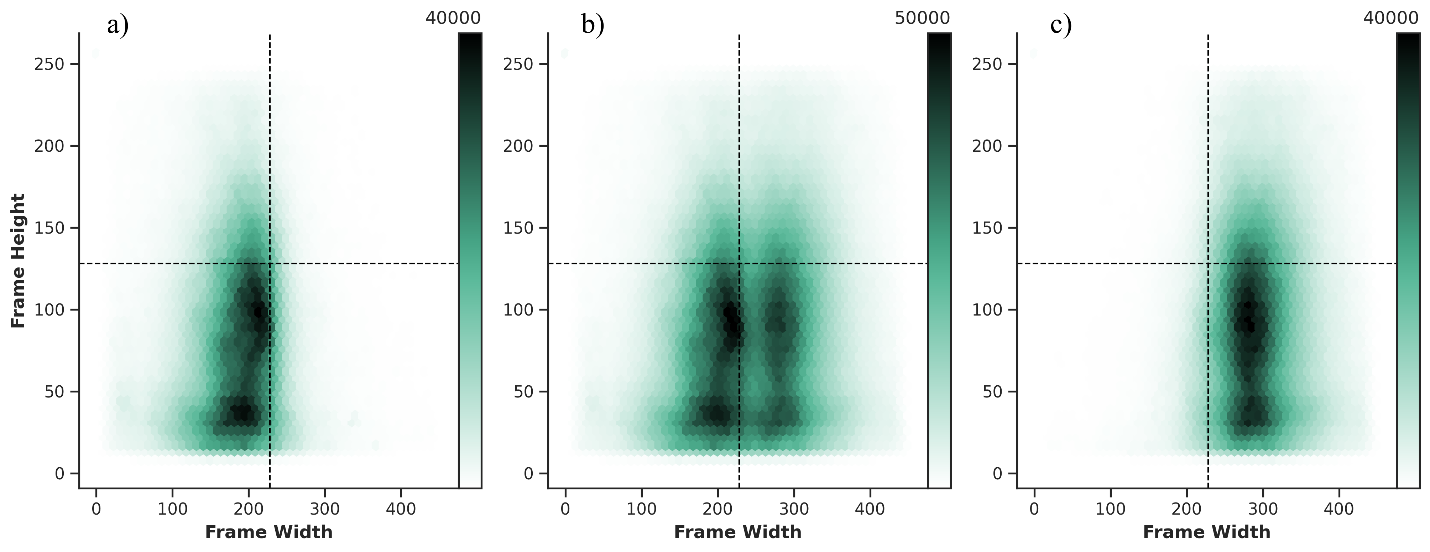

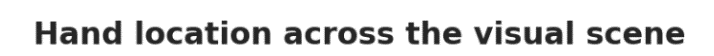


Left hand

Both hands

Right hand

**Right handers**

**Figure 2** – Hex plots showing the overall distribution of hand location for participants who were designated as left hand dominant (Total number of frames analysed = (Table 1); (a) for the left hand only, (b) irrespectively of hand (i.e., both left and right hands, for unimanual and bimanual movements) and (c) for the right hand only, across the image space. Total number of frames analysed = 31,300,201. The darkness of the colour reflects the density of the coded hand (attached colour bar indicates frame density across the visual scene), which are similarly reflected in the horizontal and vertical axis histogram bars on the top and side of the image.

**Figure 2** – Hex plots showing the overall distribution of hand location for participants who were designated as left hand dominant (Table 1); (a) for the left hand only, (b) irrespectively of hand (i.e., both left and right hands, for unimanual and bimanual movements) and (c) for the right hand only, across the image space. Total number of frames analysed = 26,966,886). The darkness of the colour reflects the density of the coded hand (attached colour bar indicates frame density across the visual scene), which are similarly reflected in the horizontal and vertical axis histogram bars on the top and side of the image.

| Hand location across image vertical space (frame %) | | | |
| --- | --- | --- | --- |
|  | | UVF | LVF |
| Total frames | Both hands | 24.0 | 76.0 |
|  | Left hand | 21.4 | 78.6 |
|  | Right hand | 26.5 | 73.5 |
|  | | | |
| Left- handers | Both hands | 25.7 | 74.3 |
|  | Left hand | 22.5 | 77.5 |
|  | Right hand | 29.1 | 70.9 |
|  | | | |
| Right- handers | Both hands | 23.8 | 76.2 |
|  | Left hand | 21.2 | 78.8 |
|  | Right hand | 26.1 | 73.9 |

Table 1 – Frame proportions across vertical hemi scenes, UVS and LVS for the totality of the participants, those participants who were designated as left- and those participants who were designated as right hand dominant (visually presented in Figures 1 and 2).

| Hand location during object interaction (frame %) | | | | | | | | |
| --- | --- | --- | --- | --- | --- | --- | --- | --- |
| Portable objects | | | |  | Stationary objects | | | |
|  | | UVF | LVF |  |  | | UVF | LVF |
| Total frames | Both hands | 25.0 | 75.0 |  | **Total frames** | Both hands | 29.1 | 70.9 |
|  | Left hand | 22.6 | 77.4 |  |  | Left hand | 24.9 | 75.1 |
|  | Right hand | 27.2 | 72.8 |  |  | Right hand | 32.9 | 67.1 |
|  | | | |  |  |  |  |  |
| Left- handers | Both hands | 27.6 | 72.4 |  | **Left- handers** | Both hands | 22.4 | 77.6 |
|  | Left hand | 24.0 | 76.0 |  |  | Left hand | 22.6 | 77.4 |
|  | Right hand | 31.7 | 68.3 |  |  | Right hand | 22.3 | 77.7 |
|  | | | |  |  |  |  |  |
| Right- handers | Both hands | 24.6 | 75.4 |  | **Right- handers** | Both hands | 30.7 | 69.3 |
|  | Left hand | 22.4 | 77.6 |  |  | Left hand | 25.3 | 64.7 |
|  | Right hand | 26.6 | 73.4 |  |  | Right hand | 35.7 | 64.3 |
|  | | | | | | | | |
| No-contact | | | |  | Self-contact | | | |
|  | | UVF | LVF |  |  | | UVF | LVF |
| Total frames | Both hands | 17.0 | 83.0 |  | **Total frames** | Both hands | 19.5 | 80.5 |
|  | Left hand | 13.8 | 86.2 |  |  | Left hand | 19.5 | 80.5 |
|  | Right hand | 20.7 | 79.3 |  |  | Right hand | 19.5 | 80.5 |
|  | | | |  |  |  |  |  |
| Left- handers | Both hands | 15.6 | 84.4 |  | **Left- handers** | Both hands | 19.1 | 80.9 |
|  | Left hand | 12.3 | 87.7 |  |  | Left hand | 18.6 | 81.4 |
|  | Right hand | 18.3 | 81.7 |  |  | Right hand | 19.6 | 80.4 |
|  | | | |  |  |  |  |  |
| Right- handers | Both hands | 17.3 | 82.7 |  | **Right- handers** | Both hands | 19.6 | 80.4 |
|  | Left hand | 14.0 | 86.0 |  |  | Left hands | 19.6 | 80.4 |
|  | Right hand | 21.2 | 78.8 |  |  | Right hands | 19.5 | 80.5 |
|  | | | | | | | | |

Table 2 – Frame proportions across vertical hemi scenes during object interaction, UVS and LVS for the totality of the participants, those participants who were designated as left- and those participants who were designated as right hand dominant (visually presented in Figures 3 and 4).


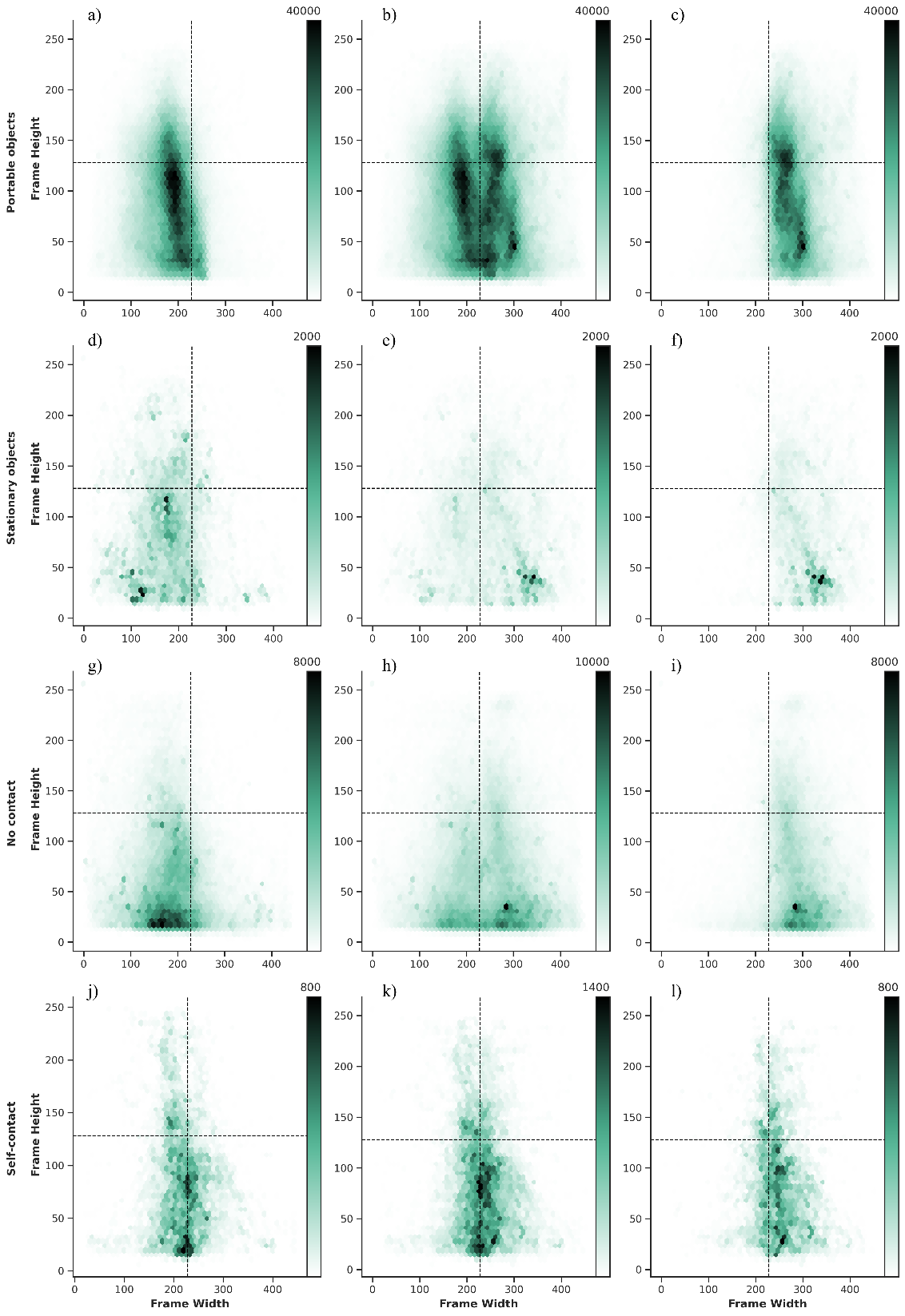

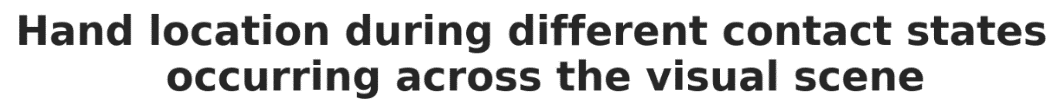


Both hands

Left hand

Right hand

**Left handers**

**Figure 3** – Hex plots representing the overall distribution of hand location during the different contact states across the visual scene for those participants designated as left hand dominant (Tables 2 and 4). On the first row, the 3-panel figure shows the overall distribution of hand location during portable object interaction, (a) for the left hand only, (b) irrespectively of hand used (d, i.e., both left and right hands, for unimanual and bimanual movements); and (c) for the right hand only, across the image space; Total number of frames analysed = 3,534,648. On the second row, the 3-panel figure shows the overall distribution of hand location during stationary object interaction, (d) for the left hand only, (e) irrespectively of hand used (d, i.e., both left and right hands, for unimanual and bimanual movements) and (f) for the right hand only, across the image space; Total number of frames analysed = 167,242. On the third row, the 3-panel figure shows the overall distribution of hand location during no object contact, (g) for the left hand only, (h) irrespectively of hand used (g, i.e., both left and right hands, for unimanual and bimanual movements) and (i) for the right hand only, across the image space; Total number of frames analysed = 582,366. On the forth row, the 3-panel figure shows the overall distribution of hand location during hand self-contact, (j) for the left hand only, (k) irrespectively of hand used (j, i.e., both left and right hands, for unimanual and bimanual movements) and (l) for the right hand only, across the image space; Total number of frames analysed = 49,059. The darkness of the colour reflects the density of the coded hand (attached colour bar indicates frame density across the visual scene), which are similarly reflected in the horizontal and vertical axis histogram bars on the top and side of the image.


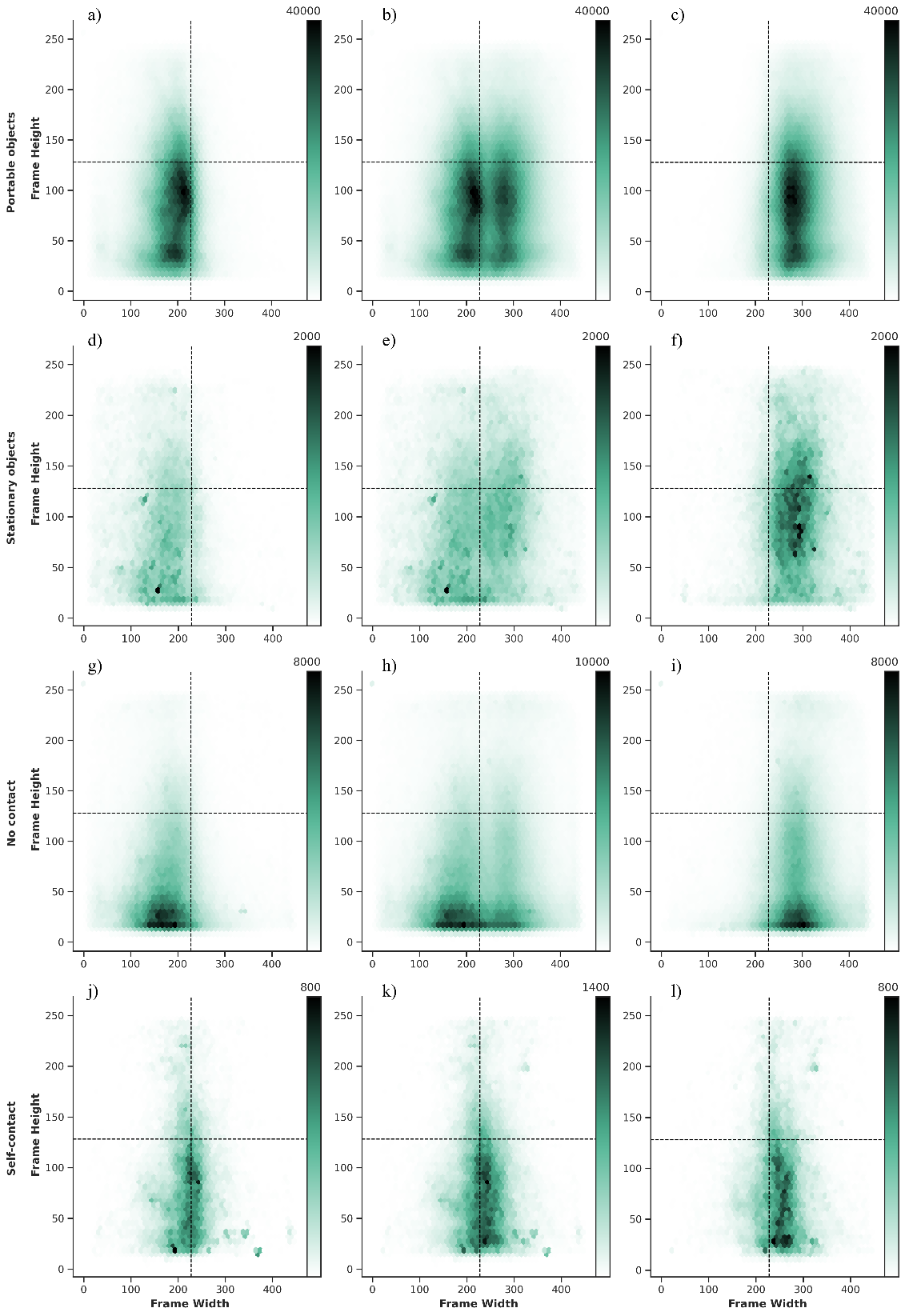

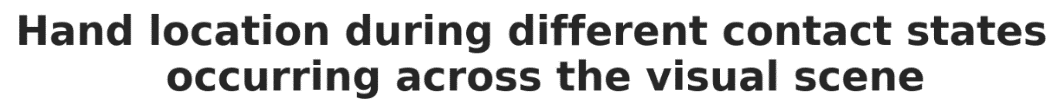


Both hands

Left hand

Right hand

**Right handers**

**Figure 4** – Hex plots representing the overall distribution of hand location during the different contact states across the visual scene for those participants designated as right hand dominant (Tables 2 and 4). On the first row, the 3-panel figure shows the overall distribution of hand location during portable object interaction, (a) for the left hand only, (b) irrespectively of hand used (d, i.e., both left and right hands, for unimanual and bimanual movements); and (c) for the right hand only, across the image space; Total number of frames analysed = 22,520,178. On the second row, the 3-panel figure shows the overall distribution of hand location during stationary object interaction, (d) for the left hand only, (e) irrespectively of hand used (d, i.e., both left and right hands, for unimanual and bimanual movements) and (f) for the right hand only, across the image space; Total number of frames analysed = 734,673. On the third row, the 3-panel figure shows the overall distribution of hand location during no object contact, (g) for the left hand only, (h) irrespectively of hand used (g, i.e., both left and right hands, for unimanual and bimanual movements) and (i) for the right hand only, across the image space; Total number of frames analysed = 3,386,497. On the forth row, the 3-panel figure shows the overall distribution of hand location during hand self-contact, (j) for the left hand only, (k) irrespectively of hand used (j, i.e., both left and right hands, for unimanual and bimanual movements) and (l) for the right hand only, across the image space; Total number of frames analysed = 325,538. The darkness of the colour reflects the density of the coded hand (attached colour bar indicates frame density across the visual scene), which are similarly reflected in the horizontal and vertical axis histogram bars on the top and side of the image.

| Hand location across visual scene quadrants  (Frame %) | | | | | |
| --- | --- | --- | --- | --- | --- |
|  | | UL | LL | UR | LR |
| Total frames | Both hands | 10.0% | 35.6% | 14.1% | 40.3% |
|  | Left hand | 18.3% | 65.9% | 3.1% | 12.7% |
|  | Right hand | 2.1% | 7.1% | 24.4 | 66.3% |
|  | | | | | |
| Left- handers | Both hands | 12.2% | 35.4% | 13.6% | 38.9% |
|  | Left hand | 20.7% | 65.5% | 1.8% | 13.9% |
|  | Right hand | 3.2% | 5.9% | 25.8% | 65.0% |
|  | | | | | |
| Right- handers | Both hands | 9.6% | 35.7% | 14.2% | 40.5% |
|  | Left hand | 17.9% | 66.3% | 3.3% | 12.4% |
|  | Right hand | 1.9% | 7.3% | 24.2% | 66.5% |

Table 3 – Frame proportions across visual scene quadrants for all the participants, those who were designated as left hand dominant and those who were designated as right hand dominant (visually presented in Figures 1 and 2).

| Hand-object interactions across visual scene quadrants (Frame %) | | | | | | | | | | | | | | | |
| --- | --- | --- | --- | --- | --- | --- | --- | --- | --- | --- | --- | --- | --- | --- | --- |
| Portable objects | | | | | | | |  | **Stationary objects** | | | | | | |
|  | | | | UL | LL | UR | LR |  |  | | UL | LL | | UR | LR |
| Total sample | | Both hands | | 10.3 | 40.0 | 14.7 | 40.4 |  | **Total frames** | Both hands | 12.1 | 34.6 | | 17.0 | 36.3 |
|  |  | Left hand | | 19.4 | 65.2 | 3.2 | 12.2 |  |  | Left hand | 21.7 | 64.5 | | 3.2 | 10.6 |
|  |  | Right hand | | 2.0 | 6.4 | 25.2 | 66.4 |  |  | Right hand | 3.4 | 7.6 | | 29.5 | 59.5 |
|  | | | | | |  |  |  |  |  |  |  | |  |  |
| Left- handers | | Both hands | | 13.3 | 35.7 | 14.3 | 36.7 |  | **Left- handers** | Both hands | 9.2 | 29.0 | | 13.2 | 48.6 |
|  |  | Left hand | | 22.2 | 63.0 | 1.8 | 13.0 |  |  | Left hand | 19.0 | 64.8 | | 3.6 | 12.6 |
|  |  | Right hand | | 3.4 | 5.2 | 28.3 | 63.1 |  |  | Right hand | 2.6 | 5.1 | | 19.7 | 72.6 |
|  | | | | | |  |  |  |  |  |  |  | |  |  |
| Right- handers | | Both hands | | 9.9 | 34.4 | 14.8 | 41.0 |  | **Right- handers** | Both hands | 12.8 | 35.9 | | 17.9 | 33.5 |
|  |  | Left hand | | 18.9 | 65.6 | 3.4 | 12.0 |  |  | Left hand | 22.2 | 64.5 | | 3.1 | 10.2 |
|  |  | Right hand | | 1.8 | 6.6 | 24.8 | 66.8 |  |  | Right hand | 3.7 | 8.3 | | 32.1 | 55.9 |
|  | | | | | | | | | | | | | | | |
| No-contact | | | | | | | |  | Self-contact | | | | | | |
|  | | | | UL | LL | UR | LR |  |  | | UL | | LL | UR | LR |
| Total sample | | Both hands | | 7.2 | 43.0 | 9.8 | 40.0 |  | **Total frames** | Both hands | 9.4 | | 34.5 | 10.1 | 46.0 |
|  |  | Left hand | | 11.6 | 72.2 | 2.2 | 14.1 |  |  | Left hand | 12.9 | | 47.2 | 6.6 | 34.3 |
|  |  | Right hand | | 2.4 | 10.5 | 18.3 | 68.8 |  |  | Right hand | 5.7 | | 22.1 | 13.9 | 58.4 |
|  | | | | | |  |  |  |  |  |  | |  |  |  |
| Left- handers | | Both hands | | 6.1 | 35.7 | 9.5 | 48.7 |  | **Left- handers** | Both hands | 10.9 | | 34.3 | 8.2 | 46.6 |
|  |  | Left hand | | 11.6 | 68.9 | 1.6 | 18.8 |  |  | Left hand | 14.4 | | 46.0 | 4.2 | 35.4 |
|  |  | Right hand | | 2.3 | 8.5 | 16.0 | 73.2 |  |  | Right hand | 7.2 | | 21.8 | 12.4 | 58.6 |
|  | | | | | |  |  |  |  |  |  | |  |  |  |
| Right- handers | | Both hands | | 7.4 | 44.3 | 9.9 | 38.5 |  | **Right- handers** | Both hands | 9.2 | | 34.5 | 10.4 | 45.9 |
|  |  | Left hand | | 11.7 | 72.7 | 2.3 | 13.4 |  |  | Left hands | 12.7 | | 46.2 | 7.0 | 34.2 |
|  |  | Right hand | | 2.4 | 10.9 | 18.8 | 67.9 |  |  | Right hands | 5.4 | | 22.1 | 14.1 | 58.4 |
|  |  | |  | | | | | | | | | | |  |  |

Table 4 – Frame proportions during object interactions across visual scene quadrants for all participants, those who were designated as left hand dominant and those who were designated as right hand dominant (visually presented in Figures 3 and 4).
